# Development and Characterization of Functional Cookies Enriched with Chestnut Shells Extract as Source of Bioactive Phenolic Compounds

**DOI:** 10.3390/foods12030640

**Published:** 2023-02-02

**Authors:** Diana Pinto, Manuela M. Moreira, Elsa F. Vieira, Jaroslava Švarc-Gajić, Anna Vallverdú-Queralt, Tanja Brezo-Borjan, Cristina Delerue-Matos, Francisca Rodrigues

**Affiliations:** 1 REQUIMTE/LAQV, ISEP, Polytechnic of Porto, rua Dr. António Bernardino de Almeida, 4249-015 Porto, Portugal; 2 Faculty of Technology, University of Novi Sad, Bulevar cara Lazara 1, 21000 Novi Sad, Serbia; 3 Nutrition, Food Science and Gastronomy Department, School of Pharmacy and Food Science, University of Barcelona, 08028 Barcelona, Spain; 4 Consorcio CIBER, M.P. Fisiopatología de la Obesidad y la Nutrición (CIBERObn), Instituto de Salud Carlos III (ISCIII), 28220 Madrid, Spain

**Keywords:** *Castanea sativa*, functional food, nutritional composition, bioactivity, phenolic compounds, sensory analysis

## Abstract

Chestnut (*Castanea sativa*) shells (CSs), an undervalued agro-industrial biowaste, have arisen as a source of bioactive compounds with promising health-promoting effects. This study attempted, for the first time, to develop a functional food, namely cookies, using a CS extract obtained by an eco-friendly technology (subcritical water extraction). The cookies were characterized regarding their nutritional composition, total phenolic and flavonoid contents (TPC and TFC, respectively), antioxidant/antiradical activities, phenolic profile, and sensory evaluation. The results demonstrated that the CS-extract-enriched cookies were mainly composed of carbohydrates (53.92% on dry weight (dw)), fat (32.62% dw), and fiber (5.15% dw). The phenolic profile outlined by HPLC-PDA revealed the presence of phenolic acids, flavonoids, and hydrolysable tannins, attesting to the high TPC and TFC. The in vitro antioxidant/antiradical effects proved the bioactivity of the functional cookies, while the sensory evaluation unveiled excellent scores on all attributes (≥6.25). The heatmap diagram corroborated strong correlations between the TPC and antioxidant/antiradical properties, predicting that the appreciated sensory attributes were closely correlated with high carbohydrates and phenolic compounds. This study encourages the sustainable recovery of antioxidants from CSs and their further employment as an active nutraceutical ingredient in functional cookies.

## 1. Introduction

The rising consumer awareness concerning the relationship between nutrition diet, health status, and wellbeing has boosted the demand for novel food products manufactured by eco-friendly technologies [1]. Therefore, functional foods have been in the spotlight owing to their commitment to nutritional needs, delivering valuable bioactive compounds that offer promissory health-promoting properties, and encompassing preventive effects against chronic diseases (such as metabolic, neurological, and cardiovascular pathologies, cancer, diabetes, and premature aging) [1,2]. Various functional foods are available on the market, including yogurts, milk, juices, bread, and cereals [2,3]. This is the same in the case of cookies, which are popular bakery products commonly prepared from wheat flour, sugar, and fat [3]. The intake of cookies has arisen as a convenient and highly consumed snack due to their pleasant taste, extended shelf life, them being ready-to-eat, and their elevated practicability for daily diets. Hence, cookies may offer a prominent opportunity as a favorable vehicle for the delivery of micronutrients (such as vitamins and minerals) and other bioactive compounds (such as phenolic compounds and carotenoids), embracing a hot research topic in the food engineering field [3].

*Castanea sativa* produces fruits (chestnuts) with a high commercial value in southern Europe [1]. Chestnut fruits are popular nuts owing to their unique sensory properties, particularly in the Mediterranean diet, with them being consumed fresh or processed into frozen nuts, flours, jams, purées, and as marron glacé [1,4]. Regarding traditional claims, the use of different parts of the *C. sativa* plant has long been documented in folk medicine to treat infertility, diarrhea, and cough [1]. The highest output of world chestnut production was estimated to be 2.3 million tons in 2020, with a production yield of 39,856 hg/ha [5]. Over the last decade, the chestnut sector has evidenced a steady growth in Europe, yielding almost 15% of the total production and contributing to the rising of chestnut by-products, mainly shells [5]. Chestnut shells (CSs) are a plentiful and underutilized agro-industrial by-product resulting from chestnut peeling, representing ≈20% of the total fruit weight [1,6]. Beyond their reuse as fuel for energy production and as a tannin source in the wine aging process, no profitable alternative applications have been proposed [1,7]. Nevertheless, ongoing studies have been centered on the discovery of new attractive applications for the valorization of CSs. The high nutritional value of CSs is ascribed to the high levels of macronutrients (namely fiber and other non-digestible oligosaccharides) and micronutrients (namely minerals, phenolic compounds, and vitamin E) that deliver outstanding health-promoting effects, including anti-inflammatory, antimicrobial, antioxidant, hypoglycemic, hypolipidemic and neuroprotective properties [1,6,8,9,10,11]. The reuse of this inexpensive raw material as source of natural antioxidants and prebiotics envisages many challenges in the design of valuable products for food and nutraceutical industries, aiming to increase the economic revenue of agro-industries, and offsetting the high-level investments of companies and governments in this field [1,10].

This notwithstanding, the use and implementation of clean and proficient extraction techniques for the isolation of bioactive molecules is still a challenge at the industrial level [12]. Alternative eco-friendly technologies, such as Subcritical Water Extraction (SWE), have emerged to accomplish a more sustainable recovery of added-value molecules, overcoming the drawbacks of conventional extraction and producing high-quality extracts with low manufacturing costs [4]. In the last two decades, several biofortification and food-to-food fortification approaches have been proposed to enhance the micronutrient content and bioavailability of cereal-derived products [3,13]. The enrichment of cookies with functional ingredients, such as phenolic compounds, could be a promising concept. Phenolic compounds may not only improve the health status of consumers but also enhance the sensory properties (e.g., appearance, taste, flavor) and functionality of products [13].

Several studies have estimated the content of bioactive compounds in isolated ingredients and natural extracts [4,6,8,12]; however, there are a lack of studies regarding the design of functional foods enriched with these bioactive molecules. This study aimed to develop cookies fortified with CS extract obtained by SWE and to characterize their nutritional composition, total phenolic and flavonoid contents (TPC and TFC, respectively), antioxidant/antiradical properties, phenolic profile, and sensory acceptance by a panel. Pearson’s correlations were outlined to investigate the contribution of the macro and micronutrients to the bioactivity and sensory properties of the cookies through a heatmap-colored diagram. To the best of our knowledge, this was the first study that attempted to develop functional cookies with a phenolic-rich CS extract.

## 2. Materials and Methods

### 2.1. Chemicals and Reagents

All chemicals and solvents used were of analytical grade, used as-received or dried by standard procedures, and obtained from commercial sources. Folin–Ciocalteu’s reagent, sodium carbonate, sodium nitrite, aluminum chloride hexahydrate, sodium hydroxide, 2,4,6-tri(2-pyridyl)-s-triazine (TPTZ), ferric chloride hexahydrate, hydrochloric acid, sodium acetate, glacial acetic acid, 2,2-diphenyl-1-picryl-hydrazyl (DPPH), sodium persulfate, 2,2′-azino-bis(3-ethylbenzothiazoline-6-sulfonic acid) diammonium salt (ABTS), β-nicotinamide adenine dinucleotide reduced dipotassium salt (NADH), nitrotetrazolium blue chloride, phenazine methosulfate (PMS), hydrogen peroxide, horseradish peroxidase, dihydrorhodamine (DHR), sodium hypochlorite, dimethylformamide (DMF), fluorescein sodium salt, sodium bicarbonate, ferrous sulfate heptahydrate, catechin, gallic acid, Trolox, and ascorbic acid were purchased from Sigma-Aldrich (Steinheim, Germany). All the remaining reagents were supplied by Sigma Chemical Co. (St. Louis, MO, USA).

For HPLC analysis, the following pure standards of phenolic acids were tested: gallic acid (≥99%), protocatechuic acid (99.63%), neochlorogenic acid (≥98%), caftaric acid (≥97%), chlorogenic acid (>95%), 4-caffeyolquinic acid (≥98%), vanillic acid (≥97%), caffeic acid (≥98%), syringic acid (≥98%), *p*-coumaric acid (≥98%), *trans*-ferulic acid (≥99%), sinapic acid (≥99%), 3,5-di-caffeyolquinic acid (≥95%), 4,5-di-*O*-caffeyolquinic acid (≥90%), cinnamic acid (≥99%); flavonoids: (+)-catechin (≥98%), (-)-epicatechin (≥90%), naringin (≥95%), quecetin-3-*O*-galactoside (≥97%), quercetin-3-*O*-glucopyranoside (≥99%), rutin hydrate (≥94%), myricetin (≥96%), quercitrin (≥97%), kaempferol-3-*O*-glucoside (≥95%), kaempferol-3-*O*-rutinoside (≥98%), isorhamnetin-3-*O*-glucoside (≥98%), isorhamnetin-3-*O*-rutinoside (≥99%), naringenin (98%), quercetin (95%), kaempferol (≥98%), apigenin (≥99%), chrysin (≥99%), tiliroside (≥98%); chalcones: phloridzin dehydrate (99%) and phloretin (≥98.5%); stilbenoids: *trans*-polydatin (≥98%), *trans*-epsilon viniferin (≥95%) and resveratrol (≥99%); hydrolysable tannin: ellagic acid (≥95%), and the alkaloid caffeine (≥95%). The standards were acquired from Sigma-Aldrich (Steinheim, Germany) and Extrasynhtese (Genay, France). Methanol (HPLC-gradient-grade) and formic acid (HPLC-gradient-grade) were obtained from Merck (Darmstadt, Germany).

### 2.2. Castanea Sativa Shells

Shells were kindly supplied by Sortegel (Sortes, Bragança, Portugal) in October 2018. After dehydration at 40 °C for 24 h, CSs were ground to fine particle size (≈1 mm) using an ultra-centrifugal grinder (Retsch ZM200, Germany). The powdered samples were stored at room temperature in the dark until extraction.

### 2.3. Preparation of Chestnut Shells Extract by Subcritical Water Extraction

Chestnut shells were extracted by SWE according to Švarc-Gajić et al. [14].

### 2.4. Preparation of Cookies Enriched with Chestnut Shells Extract

The formulation of the cookies in this study is presented in Table 1. Briefly, white flour, sugar, cocoa powder, lyophilized CS extract, sodium bicarbonate, and sodium chloride were mixed to form a homogeneous mixture. Then, melted butter and eggs were added. The cookies were formed with a 1 cm thickness and a 5 cm diameter. The cookies were sprinkled with chocolate chips and then baked at 160 °C for 30 min and allowed to cool. Control cookies formulated without extract were also prepared. After this, the cookies were packed in airtight laminated pouches and stored at ambient temperature for further analysis.

### 2.5. Approximate Composition

The nutritional composition of the cookies was evaluated regarding their carbohydrate, fat, protein, moisture, ash, and fiber contents. Moisture was estimated at 105 °C until a constant weight using a moisture balance (KERN MLS50 160–3 Infra-Red analyzer). Protein content was analyzed by the Kjeldahl method to determine the total nitrogen by applying a conversion factor of 6.25 [15]. Fat and ash were assessed following the AOAC methods [15], while the carbohydrate content was determined by the difference between the other macronutrients. Fiber content was estimated using a commercial kit (Sigma), according to the manufacturer’s instructions. The approximate composition was presented as a percentage of dry weight (% dw). Total energy, in kilocalories, was calculated according to the following Equation (1):

Energy (kcal/100 g) = 4 × (g proteins + g carbohydrates) + 9 × (g fat) (1)

The total energy, in kilojoules, was then calculated by multiplying the kilocalories by a conversion factor of 4.2.

### 2.6. Extraction of Bioactive Compounds from Cookies

The extraction of bioactive compounds was accomplished following the procedure described by Najjar et al. [16], with minor modifications. The cookies were ground in a Moulinex A320 grinder (France). For the extraction, crushed cookies (1 g) were mixed with 5 mL of distilled water, extracted in a sonicator at 40 °C for 5 min, maintained at 4 °C for 30 min, centrifuged at 10,000× *g* for 10 min, and the supernatant was collected. Then, the solid residue was re-extracted with 2 mL of distilled water, sonicated at 40 °C for 15 min, kept for 30 min at 4 °C, and centrifuged at 10,000× *g* for 10 min. Finally, the supernatants were assembled and stored at −20 °C until analysis. The extraction was performed in triplicate.

### 2.7. Total Phenolic and Flavonoid Contents and In Vitro Bioactivity

The TPC and TFC were screened according to the methods validated by Pinto et al. [10]. TPC results were presented as mg of gallic acid equivalents (GAE) per 100 g of cookies (mg GAE/100 g cookies). TFC was presented as mg of catechin equivalents (CE) per 100 g of cookies (mg CE/100 g cookies). The in vitro antioxidant and antiradical activities were evaluated by a ferric reducing antioxidant power (FRAP) assay and DPPH and ABTS radical scavenging assays [12]. FRAP values were expressed in mg of ferrous sulfate equivalents (FSE) per 100 g of cookies (mg FSE/100 g cookies). The DPPH and ABTS results were presented in mg of Trolox equivalents (TE) and mg of ascorbic acid equivalents (AAE), respectively, per 100 g of cookies (mg TE/100 g cookies and mg AAE/100 g cookies).

### 2.8. Reactive-Oxygen- and Nitrogen-Species-Counteracting Activity

The potential of the bioactive extracts from the cookies enriched with CS extract in quenching reactive oxygen and nitrogen species (ROS and RNS, respectively) was assessed using a Synergy HT Microplate Reader with a thermostat (BioTek Instruments, Inc., Winooski, VT, USA) as described by Pinto et al. [17]. Catechin and gallic acid were employed as positive controls.

#### 2.8.1. Superoxide Anion Radical Quenching Assay

The superoxide-anion-radical (O_2_^●−^)-quenching capacity was assessed through the amount of purple-colored diformazan formed from the NBT reduction induced by a non-enzymatic NADH/PMS/O_2_ system used to generate O_2_^●−^ following the procedure validated by Pinto et al. [17]. Absorbance was read at 560 nm for 5 min, and the results were expressed as the inhibition, in % or IC_50_ (µg/mL).

#### 2.8.2. Hydrogen Peroxide Quenching Assay

The hydrogen-peroxide (H_2_O_2_)-quenching capacity was determined by the extent of ABTS oxidation induced by H_2_O_2_ and catalyzed by peroxidase according to Pinto et al. [17]. The results were presented as the inhibition, in % or IC_50_ (µg/mL), after measuring the absorbance at 405 nm.

#### 2.8.3. Hypochlorous Acid Quenching Assay

The quenching capacity against hypochlorous acid (HOCl) was appraised based on the oxidation of DHR to rhodamine induced by HOCl following the methodology validated by Pinto et al. [17]. The fluorescence was monitored at 37 °C for 5 min, and the results were presented as the inhibition, in % or IC_50_ (µg/mL).

#### 2.8.4. Peroxyl Radical Quenching Assay

The peroxyl-radical (ROO^●^)-quenching ability was estimated through an oxygen radical absorbance capacity (ORAC) assay by the oxidation of fluorescein induced by ROO^●^, causing the fluorescence decay monitored at 37 °C for 2 h [17]. Trolox was used as the standard. The results were expressed as µg of TE per mg cookies.

#### 2.8.5. Peroxynitrite Quenching Assay

The peroxynitrite (ONOO^−^)-quenching potential was ascertained according to Pinto et al. [17] based on the oxidation of non-fluorescent DHR to fluorescent rhodamine by ONOO^−^ in the absence and presence of 25 mM sodium bicarbonate to simulate the physiological CO_2_ levels. The results were presented as the inhibition, in % or IC_50_ (µg/mL).

### 2.9. Phenolic Composition by HPLC-PDA

The phenolic composition was investigated by a Shimadzu HPLC system equipped with a CTO-10AS VP column oven, a DGU-20AS prominence degasser, an LC-20AD prominence pump, an SIL-20A HT prominence autosampler, and an SPD-M20A photodiode array (PDA) detector (Kyoto, Japan), following the methodology validated by Moreira et al. [18]. The results were expressed as mg of each phenolic compound per 100 g of cookies (mg/100 g cookies).

### 2.10. Sensory Evaluation

The sensory evaluation was conducted by a panel of twenty volunteers with previous experience in sensory analysis. The sensory tests were designed following international standards [19,20]. Regarding the panelists, fourteen were women (average age = 36 years) and six were men (average age = 27 years). The panelists evaluated a set of nine attributes, including appearance, color, texture, taste, flavor, sweetness, crunchiness, hardness, and overall acceptability, using a nine-point hedonic scale (1 = “disliked extremely”, 9 = “liked extremely”). Cookies were served to the volunteers on dispensable plates and were accompanied by water to minimize carry-over effects from the previous food consumed. The consent of all volunteers was obtained before the sensory tests. The questionnaire was anonymous. A separate question was included to ascertain if the volunteers would buy the functional cookies (yes/no). For this analysis, the following inclusion criteria were considered: (i) usually consume this type of product; (ii) absence of health problems that may affect the sensory test; and (iii) absence of any food allergy that could affect the volunteer’s health (e.g., nut allergy).

### 2.11. Statistical Analysis

Data were presented as mean ± standard deviation of at least three independent experiments. The statistical differences were investigated by one-way ANOVA and post hoc comparisons of the means through Tukey’s HSD test using the IBM SPSS Statistics 24.0 software (SPSS Inc., Chicago, IL, USA). A significant result was denoted for *p* < 0.05. Pearson’s correlation coefficients ‘*r*^2′^ were determined using the R software version 4.2.1 (R Core Team, USA) to disclose the contribution of macro and micronutrients to the bioactivity and sensory properties of the functional cookies. The correlations were presented in a heatmap-colored diagram. For the in vitro radical scavenging assays, the curves of the inhibition percentage versus the extract concentration were plotted using GraphPad Prism 9 software (La Jolla, CA, USA), and the IC_50_ values were calculated as the concentration needed to achieve 50% of the inhibition capacity.

## 3. Results and Discussion

### 3.1. Approximate Composition of Functional Cookies

The assessment of the nutritional composition is a reliable indicator of the quality, maturation state, and storage of fruits and their by-products, directly influencing their commercial value. Table 2 summarizes the approximate composition of the functional cookies enriched with CS extract and the control cookies (without extract).

According to Table 2, the functional cookies were mainly composed of carbohydrates (53.92% dw) and fat (32.62% dw). The cookies fortified with CS extract presented a moderate content of fiber (5.15% dw) and minerals (2.14% dw of ash), while low amounts of protein (6.17% dw) and moisture (4.77%) were determined. A similar nutritional composition (*p* < 0.05) was determined for the control cookies formulated without extract, apart from the fat and ash contents that achieved significant differences (*p* > 0.05) when compared to the functional cookies. The use of ingredients from different batches to prepare the functional cookies and control cookies may explain the slight differences in the fat and ash contents observed.

A previous study from Rodrigues et al. [8] showed promising results for the valorization of this by-product. The authors reported high levels of carbohydrates (56.51–74.06% dw), moisture (21.29–38.61% dw), and minerals (1.08–1.60% dw) in CSs from different Portuguese producing regions (namely Minho, Trás-os-Montes, and Beira-Alta), while low protein (2.77–3.13% dw) and fat contents (0.15–0.52% dw) were reported.

The present results were in accordance with the ones published by Nakov et al. [21], who achieved similar contents of carbohydrates (65.03–71.25% dw), protein (6.03–7.02% dw), and moisture (4.61–5.99%) in cookies fortified with increasing amounts of hulless barley flour (0–100%), while lower levels of fat (≈15%) and minerals (0.78–1.47% dw) were detected. The total dietary fiber content ranged from 3.50 to 9.84% dw, of which the highest portion corresponded to insoluble fiber (2.13–5.24% dw). Additionally, the energy value was identical, ranging from 1861 (without hulless barley flour) to 1791 kJ/100 g of cookies (with 100% hulless barley flour) [21]. In another study, Filipović et al. [22] formulated cookies enriched with different percentages (2.5–25% dw) of lyophilized and dehydrated peach using a similar formula to the present work and compared them with a control cookie (without peach). A similar nutritional profile was described for the functional cookies, with 5.41–5.67% dw protein, 69.68–70.16% dw carbohydrates, 9.91–10.84% dw fat, and 0.42–0.74% dw ash, compared to the control cookie (protein: 5.37% dw; carbohydrates: 69.49% dw; fat: 10.97% dw; ash: 0.35% dw). Moreover, Desai et al. [23] also valorized a coffee by-product, namely roasted and unroasted spent green coffee, as a functional ingredient for cookies by incorporating different percentages (3, 6, 10, and 12%). The moisture and mineral contents were comparable to the ones obtained in the present study, while substantially lower fat (1.25–2.78%) and higher protein contents (11.87–14.35%) were detected.

### 3.2. Phenolic Content and In Vitro Bioactivity

Table 3 summarizes the TPC, TFC, and antioxidant/antiradical results of the functional cookies enriched with CS extract and the control cookies (without extract).

As shown in Table 3, the functional cookies achieved a TPC of 163.53 mg GAE/100 g of cookies. As expected, this result was significantly higher (*p* < 0.05) than the one determined for the control cookies without extract (55.89 mg GAE/100 g cookies). The TPC of the functional cookies was also lower than the one reported for the CS extract prepared by SWE [24] and used to formulate the cookies (122.31–239.53 mg GAE/g dw). Nevertheless, similar outcomes were described by Uriarte-Frías et al. [25] for functional cookies prepared with flours from oyster mushroom, nopal, and amaranth (677.6–1366 µg GAE/g cookies) compared to a control cookie prepared with wheat flour (272.1 µg GAE/g cookies). This value was also in line with the one reported for spent-coffee-grounds-enriched cookies (174.4 mg GAE/100 g), whose TPC was also lower than the TPC of the spent coffee grounds used to prepare the cookies (1067.2 mg GAE/100 g) [26]. Oppositely, a substantially lower TPC was described for cookies prepared with increasing amounts of hulless barley flour (160.53–621.42 µg GAE/g dw from 0 to 100% flour) and for cookies containing dehydrated peach (9.31–43.38 mg GAE/100 g) [21,22].

Flavonoids are a representative class of phenolic compounds with remarkable bioactivity on chronic venous insufficiency by upgrading the resistance of blood vessels and improving the microvascular blood flow [1]. The functional cookies fortified with CS extract disclosed a high TFC (51.49 mg CE/100 g cookies), supporting the idea that the cookies may deliver considerable amounts of flavonoids to consumers. This result was also significantly higher (*p* < 0.05) than the TFC obtained for the control cookies (16.31 mg CE/100 g cookies). Recently, Uriarte-Frías et al. [25] reported significantly lower value of TFC in cookies prepared with a mixture of alternative vegetable flours (9.97–64.72 µg epicatechin equivalents (ECE)/g dw versus 11.25 µg ECE/g dw for a control cookie).

The antioxidant and antiradical activities of the formulated cookies were evaluated by different assays with distinctive mechanisms, namely the reduction of ionic complexes (i.e., Fe^3+^-TPTZ complex reduced to Fe^2+^-TPTZ by ferric reducing antioxidant power (FRAP)) and the scavenging of synthetic (DPPH^•^ and ABTS^•+^) radicals. As stated in Table 3, the CS-extract-enriched cookies were revealed to be potent scavengers of ABTS^•+^ and DPPH^•^ radicals, achieving results of 146.59 mg AAE/100 g cookies and 67.24 mg TE/100 g cookies, respectively. Additionally, the high antioxidant potential was proved by the FRAP result (730.16 mg FSE/100 g cookies). Compared to the control cookies formulated without extract, a significantly higher (*p* < 0.05) antioxidant capacity was attested for the functional cookies enriched with CS extract regarding the ABTS, DPPH, and FRAP assays. These results were better than the ones obtained by Filipović et al. [22] for cookies fortified with 2.5% to 25% of dehydrated peach (DPPH: 0.15–1.00 µmol TE/100 g versus 0.08 µmol TE/100 g for control cookies; ABTS: 7.42–140.14 µmol TE/100 g versus 2.53 µmol TE/100 g for control cookies), even though the lowest amount of dehydrated peach added to these cookies (2.5%) was twice as high as the amount of CS extract used in the cookies formulated in this study (1.77%). Likewise, similar outcomes were determined for cookies enriched with 20% of olive pomace (0.69–1.84, 1.04–2.85, and 22.71–68.30 µmol TE/g cookies, respectively, for ABTS, DPPH, and FRAP assays), this being twelve times higher than the amount of CS extract added to the cookies prepared in this study [27]. In addition to the lower TPC (52.3–112.9 mg GAE/100 g dw), Giuberti et al. [13] also reported a lower antioxidant activity in a FRAP assay (66.4–194.6 mg GAE/100 g dw) for gluten-free cookies formulated with 15% to 45% of alfalfa seed flour. These discrepancies may be explained by the high amounts of alternative flours used when compared to the CS extract. In contrast, slightly higher antioxidant properties were reported for cookies formulated with uncommon cereal flours used as alternatives to wheat flour (including whole spelt, oat flakes, barley flakes, kamut, and red wheat), edible flowers (namely lavender, rose, hop flower, jasmine, and elderflower), and dried fruit flours (including apricot, raspberry, strawberry, cashew, malt extract, ginger, mango, matcha tea, lemon peel, and apple) [28]. The ABTS results ranged between 3.11 and 3.45 mg AAE/g versus 1.25 mg AAE/g for the control cookies (formulated with wheat flour without adding flowers and fruits), while the DPPH results varied from 1.36 to 2.10 mg TE/g versus 0.47 mg TE/g by the control [28]. The higher results obtained in the previous study may be ascribed to the use of several plant-derived active ingredients to prepare the cookies, which may lead to synergistic effects between the different antioxidants present in each plant material comparatively to the present study.

It is worth noting that the outstanding antioxidant and antiradical properties of the functional cookies enriched with CS extract were probably attributed to the phenolic compounds recovered that have been proposed as effective natural antioxidants with recognized health-promoting effects and benefits in terms of extending the shelf-life of food products due to their antimicrobial properties and their prevention of lipid oxidation [1,4,12]. Beyond the well-documented in vitro bioactivity [4,9], a recent study also proved the in vivo antioxidant effects of CS extract prepared by SWE by upregulating the activity of antioxidant enzymes and downregulating lipid peroxidation, suggesting its use as a prominent nutraceutical ingredient [11]. In addition, the authors underlined the mild hypolipidemic and hypoglycemic effects of the CS extract [11].

### 3.3. Reactive-Oxygen- and Nitrogen-Species-Counteracting Potential

The rising awareness of society about chronic diseases and premature aging has forced industries to find solutions to prevent the impairment of health and delay the signs of aging. Simultaneously, the concern regarding sustainability issues has encouraged the search for natural products containing bioactive ingredients extracted from plant-derived materials. The protective effects against oxidative-stress-mediated damages on biomolecules (i.e., lipids, proteins, and DNA) encourage the employment of natural antioxidants as food additives and nutraceuticals, aiming to ameliorate physical- and mental-health-related disorders owing to their anti-aging effects. Agro-industrial by-products (such as CS) are exceptional sources of natural antioxidants, namely phenolic compounds and vitamin E. Table 4 summarizes the radical-oxygen- and nitrogen-species (ROS and RNS, respectively)-scavenging ability of the developed functional and control cookies.

As shown in Table 4, the CS-extract-enriched cookies achieved the highest scavenging efficiency against HOCl (IC_50_ = 81.81 µg/mL), followed by ONOO^−^, in the absence and presence of NaHCO_3_ (IC_50_ = 115.00 and 108.15 µg/mL, respectively). Furthermore, a high counteracting power was also attained for H_2_O_2_ (28.23% inhibition), O_2_^●−^ (35.85% inhibition), and ROO^●^ (5.81 µg TE/mg dw). Oxidative stress status induces a cascade of increasingly powerful and harmful reactive species initiated by the production of O_2_^●−^, which are further converted into H_2_O_2_ by superoxide dismutase, generating more toxic species such as HO^●^ and HOCl [10]. The results demonstrated that gallic acid was the best O_2_^●−^ scavenger (IC_50_ = 10.95 µg/mL), followed by catechin (IC_50_ = 48.21 µg/mL), the cookies enriched with CS extract (35.85% inhibition), and the control cookies without extract (33.41% inhibition). The functional cookies and positive controls showed significantly different results (*p* < 0.05). However, no significant differences (*p* > 0.05) were attained between the cookies formulated with CS extract and the control cookies. Recently, Ferreira et al. [24] appraised the in vitro radical-quenching ability of CS extracts prepared by SWE under similar extraction conditions (110–180 °C) to the extract used in this study to enrich cookies. As expected, these extracts achieved a substantially higher potential to scavenge O_2_^●−^ (IC_50_ values ranging from 31.14 to 73.18 µg/mL for 140 °C and 180 °C, respectively), even though gallic acid, used as a positive control, attained better results (IC_50_ = 23.82 µg/mL). Likewise, Pinto et al. [4] also described better results (IC_50_ = 12.92 µg/mL) for a CS extract prepared by the same extraction technology at a higher temperature (220 °C/30 min).

The H_2_O_2_-quenching ability was enhanced in the following order: control cookies (13.39% inhibition) < CS-extract-enriched cookies (28.23% inhibition) < gallic acid (IC_50_ = 106.03 µg/mL) < catechin (IC_50_ = 20.78 µg/mL). The counteracting efficiency of the functional cookies was significantly different (*p* < 0.05) from the control cookies (without extract), catechin, and gallic acid. For instance, Squillaci et al. [29] investigated the ROS-scavenging potential of CS aqueous extracts by exposing keratinocytes (HaCaT) to 450 µM H_2_O_2_ for 1 h. The extracts prepared from a mixture of chestnut inner and outer shells achieved around a 70% H_2_O_2_ inhibition at concentrations of 0.0004% and 0.002%.

Considering the HOCl quenching assay, a higher result was disclosed by the functional cookies, with an IC_50_ of 81.81 µg/mL, while catechin and gallic acid revealed lower IC_50_ values (0.37 and 1.81 µg/mL, respectively). The control cookies (without extract) had a lower capacity to scavenge HOCl (47.00% inhibition), since it was not possible to determine the IC_50_, which was opposite to the functional cookies enriched with CS extract. The results achieved by the cookies and the positive controls were significantly different (*p* < 0.05). Likewise, the HOCl-scavenging potential of the functional cookies and the control cookies was also significantly different (*p* < 0.05). Ferreira et al. [24] and Pinto et al. [4] unveiled a better HOCl-scavenging power for eco-friendly CS extracts prepared by SWE at different temperatures (110–180 °C and 230 °C) for 30 min, reporting IC_50_ values of 4.47–22.85 µg/mL and 0.79 µg/mL, respectively.

Through the ORAC assay, the prevention of lipid peroxidation by antioxidants was explored based on the counteracting activity against ROO^●^. The outcomes demonstrated that catechin was the best ROO^●^ quencher (368.95 µg TE/mg dw), followed by gallic acid (247.09 µg TE/mg dw), the functional cookies enriched with CS extract (5.81 µg TE/mg dw), and the control cookies without extract (0.76 µg TE/mg dw). Significant differences (*p* < 0.05) were attained between the functional cookies, the control cookies, gallic acid, and catechin. Previous studies reported higher results for phenolics-rich CS extracts recovered by the same extraction technology employed in this study (0.32 µmol TE/mg dw and 0.49–0.73 µmol TE/mg dw) [4,24].

The ONOO^−^ quenching assay was accomplished in the absence and presence of sodium bicarbonate to simulate the physiological concentrations of bicarbonate (≈25 mM) that may induce a considerable drop in the scavenging potential of certain polyphenols (such as caffeic, ferulic, gallic, and *p*-coumaric acids) oppositely to others (for example, catechin and derivatives) regarding ONOO^−^-mediated tyrosine nitration [10]. Considering the results, a lower concentration of CS-extract-enriched cookies was needed to scavenge 50% of the ONOO^−^ formed in the tested medium containing NaHCO_3_ (IC_50_ = 108.15 µg/mL) than in its absence (IC_50_ = 115.00 µg/mL). As expected, the functional cookies displayed a substantially better ONOO^−^-scavenging potential when compared to the control cookies, since it was not possible to determine the IC_50_ for the control cookies, with the results being expressed as the inhibition percentage (45.56% and 38.48% inhibition, respectively, in the presence and absence of NaHCO_3_). Conversely, a lower concentration of gallic acid and catechin was required to inhibit half of the ONOO^−^ formed in the tested medium without NaHCO_3_ (IC_50_ of 0.15 and 0.16 µg/mL, respectively) than in its presence (IC_50_ of 0.27 and 0.23 µg/mL, respectively). The cookies disclosed a significantly different IC_50_ (*p* < 0.05) value when compared to catechin and gallic acid that showed similar results (*p* > 0.05). In addition, only the control cookies (without extract) and the functional cookies displayed significantly different (*p* < 0.05) results in the presence and absence of NaHCO_3_.

The promising outcomes of the radical-quenching capacity of the cookies fortified with CS extract were probably due to their phytochemical composition, particularly their richness in phenolic acids (mainly gallic acid and caffeoylquinic acids derivatives), flavonoids (mainly catechin), and hydrolysable tannins (namely ellagic acid), whose scavenging potential has already been proven in previous studies [30,31,32]. Notably, the cookies enriched with CS extract disclosed, in general, a markedly higher counteracting power when compared to the control cookies (without extract), emphasizing the exceptional contribution of the CS extract to the radical-scavenging capacity of the functional cookies.

### 3.4. Phenolic Composition of Functional Cookies

**Figure 1:**
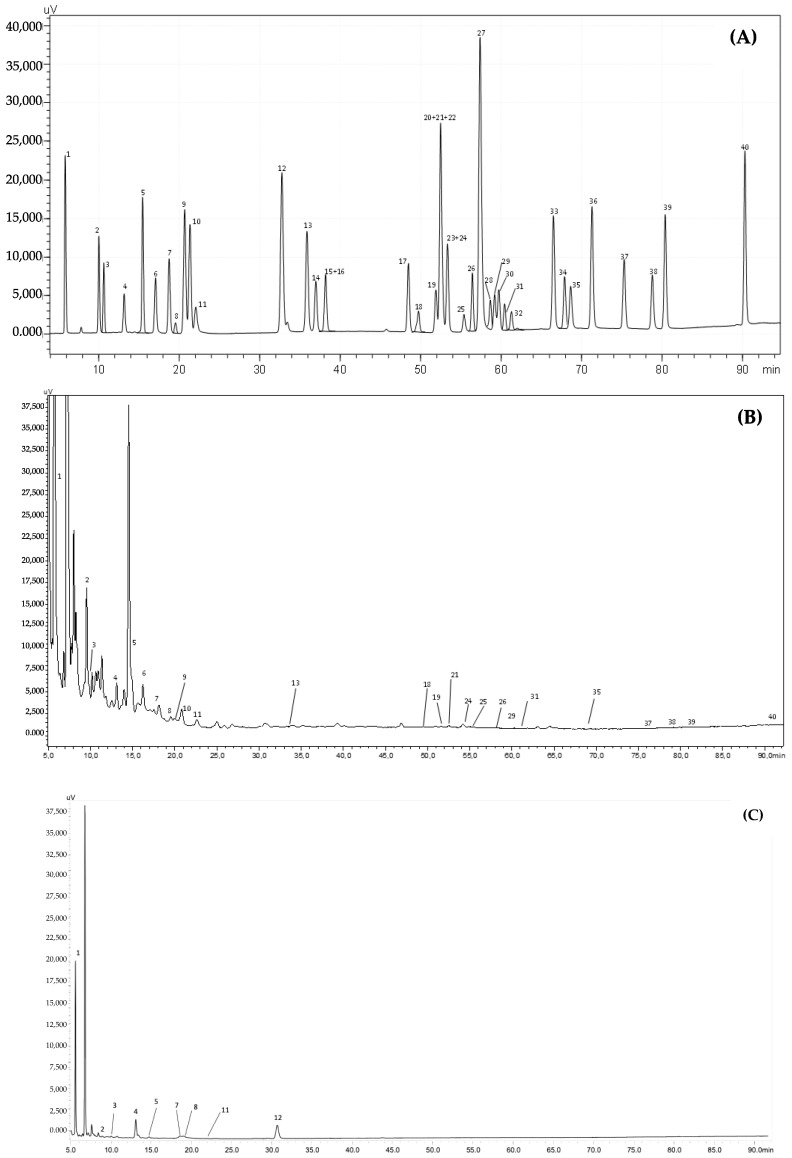
HPLC-PDA chromatogram monitored at 280 nm for (**A**) polyphenol standard mixture of 5 mg/L, (**B**) functional cookie extract, and (**C**) control cookie extract. Peak identification: (1) gallic acid, (2) protocatechuic acid, (3) neochlorogenic acid, (4) (+)-catechin, (5) caftaric acid, (6) caffeine, (7) chlorogenic acid, (8) 4-*O*-caffeyolquinic acid, (9) vanillic acid, (10) caffeic acid, (11) syringic acid, (12) (−)-epicatechin, (13) *p*-coumaric acid, (14) *trans*-ferulic acid, (15) sinapic acid, (16) *trans*-polydatin, (17) naringin, (18) 3,5-di-caffeoylquinic acid, (19) quercetin-3-*O*-galactoside, (20) resveratrol, (21) quercetin-3-*O*-glucopyranoside, (22) rutin, (23) phloridzin, (24) ellagic acid, (25) 3,4-di-*O*-caffeoylquinic acid; (26) myricetin, (27) cinnamic acid, (28) quercitrin, (29) kaempferol-3-*O*-glucoside, (30) isorhamnetin-3-*O*-glucoside, (31) kaempferol-3-*O*-rutinoside, (32) isorhamnetin-3-*O*-rutinoside, (33) naringenin, (34) *trans*-epsilon viniferin, (35) quercetin, (36) phloretin, (37) tiliroside, (38) kaempferol, (39) apigenin, and (40) chrysin.

Natural antioxidants recovered from agro-industrial residues, usually discarded as waste, have been explored with proficient and clean techniques. Beyond their remarkable health-promoting properties and effectiveness in preventing cellular damage triggered by oxidative stress, phenolics-rich extracts have been employed by the food industry as natural preservatives to replace synthetic ones that are considered toxic (e.g., butylated hydroxytoluene, butylated hydroxyanisole, and *tert*-butylhydroquinone), prevent lipid oxidation and microorganism growth, and preserve the nutritional and sensory properties of foods. Figure 1 depicts examples of HPLC-PDA chromatograms obtained for the standards and cookies. Table 5 presents the phenolic composition of the functional cookies enriched with CS extract and the control cookies (without extract).

A total of 25 phenolic compounds were identified in the functional cookies enriched with CS extract, corresponding to 154.09 mg/100 g cookies. Phenolic acids (103.41 mg/100 g cookies) were the major polyphenolic class identified, representing 67% of the total phenolic content. The predominant phenolic compounds detected in the functional cookies were gallic acid (89.4 mg/100 g cookies), ellagic acid (40.0 mg/100 g cookies), and catechin (5.17 mg/100 g cookies). Among the phenolic acids, 89% corresponded to hydroxybenzoic acids and 11% corresponded to hydroxycinnamic acids. Four hydroxybenzoic acids were found (namely gallic acid, protocatechuic acid, syringic acid, and vanillic acid), while eight hydroxycinnamic acids were identified, including caffeoylquinic acids and derivatives (5.39 mg/100 g cookies), chlorogenic acid (2.68 mg/100 g cookies), and caffeic acid (0.79 mg/100 g cookies). Ellagic acid (40.0 mg/100 g cookies) was the only hydrolysable tannin identified, representing more than 25% of the total content. Moreover, flavonoids contributed to only 5% of the total content, with flavanols being the most abundant subclass (5.17 mg/100 g cookies), followed by flavonols (2.78 mg/100 g cookies) and flavones (0.085 mg/100 g cookies). Among the flavonoids, catechin, quercetin derivatives (1.49 mg/100 g cookies), and kaempferol derivatives (0.91 mg/100 g cookies) were the most representative compounds. Myricetin (0.36 mg/100 g cookies) and tiliroside (0.018 mg/100 g cookies) were also detected in trace levels. In addition, considerable amounts of caffeine (2.71 mg/100 g cookies) were determined.

Conversely, only eight phenolic compounds were identified in the control cookies. Phenolic acids represented 85% of the total phenolic content, while the remaining 15% corresponded to flavonoids. In contrast to the functional cookies, no hydrolysable tannins, flavonols, flavones, or caffeine were detected in the control cookies. Gallic acid (36.5 mg/100 g cookies) was the major phenolic compound detected in the control cookies, followed by catechin (4.64 mg/100 g cookies). Although ellagic acid was one of the main phenolic compounds in the functional cookies, this compound was not identified in the control cookies. As expected, the total content of phenolic compounds in the control cookies (52.66 mg/100 g cookies) was significantly lower than that observed in the functional cookies, which was probably due to the absence of the phenolics-rich CS extract in their composition. The source of the phenolic compounds identified in the control cookies formulated without CS extract was possibly a consequence of the remaining ingredients, particularly cacao powder, whose phenolic composition has been extensively studied, with the presence of gallic acid, protocatechuic acid, chlorogenic acid, catechin, epicatechin, and caffeine being reported in cacao powder [33,34].

Previous studies have characterized the phytochemical profile of CS extracts prepared by conventional and green technologies, such as SWE, supercritical fluid extraction (SFE), ultrasound-assisted extraction (UAE), and microwave-assisted extraction (MAE) [4,7,8,10,12]. In a very recent study, Ferreira et al. [24] reported an identical phenolic composition in CS extract prepared by SWE under similar extraction conditions (110–180 °C for 30 min). Catechin (0.69–4.10 mg/g dw) and gallic acid (2.40–5.99 mg/g dw) were identified as the major phenolic compounds, while caffeine (0.44–1.18 mg/g dw) was also detected in substantial levels. The extract prepared at 110 °C was revealed to be richer in phenolic compounds, with a total of 20.20 mg/g dw. Likewise, Pinto et al. [4] reported the presence of ellagic acid (2.86 mg/g dw), protocatechuic acid (2.83 mg/g dw), pyrogallol (2.39 mg/g dw), methyl gallate (0.77 mg/g dw), and gallic acid (0.54 mg/g dw) in CS extract prepared by SWE at 220 °C/30 min. Other studies have also described the presence of phenolic acids (i.e., gallic acid, caffeic acid, and derivatives), flavonoids (i.e., catechin, epicatechin, epigallocatechin, apigenin, luteolin, myricetin, and quercetin derivatives), anthocyanins (i.e., proanthocyanidins and procyanidins polymers), and tannins, including hydrolysable (such as ellagic acid) and condensed (such as vescalagin and castalagin) tannins in CS extracts prepared by the SFE, UAE, and MAE techniques [7,10,12]. In general, the outcomes for the phenolic composition of the functional cookies agreed with the previous research on CS extracts. This notwithstanding, lower amounts of phenolic compounds were determined in the cookies when compared to the CS extract. To the best of our knowledge, this was the first study that formulated a functional food enriched with a chestnut by-product and that provided a comprehensive assessment of the phenolic composition, encompassing a novelty in the food and nutraceutical fields.

### 3.5. Sensory Evaluation of Functional Cookies

**Figure 2:**
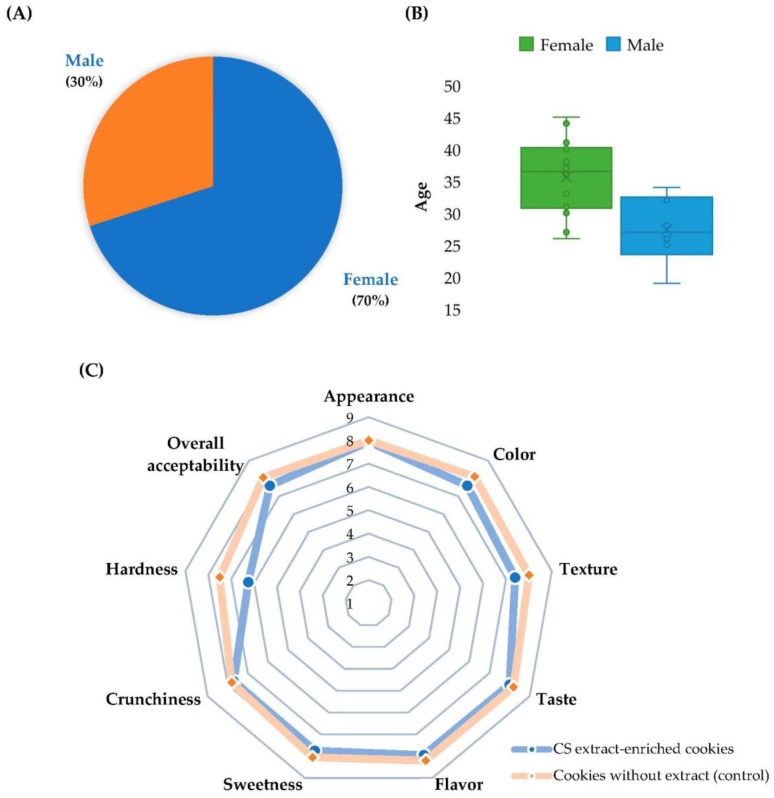
Sensory evaluation of functional cookies enriched with chestnut shells extract and control cookies (without extract). (**A**) Panelists by gender; (**B**) panelists by age; (**C**) scores of sensory attributes evaluated.

Sensory testing evaluates the attributes of a food product through the five senses (i.e., hearing, sight, smell, taste, and touch), allowing a consumer-oriented design of new products. The nine-point hedonic scale is an effective and simple categoric measuring tool that is widely used to explore sensory differences among beverages or foods and is frequently used to predict the acceptance of novel food products by potential consumers. Among the advantages, this hedonic technique: (i) is easy to use for participants compared to other scaling methodologies (e.g., estimation of magnitude), (ii) has no need for the extensive training of participants, (iii) involves easier data handling than other techniques assessing fractions (e.g., measuring lines and recording magnitude estimation), and (iv) has an identical sensitivity to other scaling techniques (e.g., line marking and magnitude estimation) regarding its discrimination power. The sensory properties of the functional cookies enriched with CS extract were assessed using a nine-point hedonic scale. The results are depicted in Figure 2.

Among the panelists, 70% were women with an age interval from 26 to 45 years old (average age = 36 years), while 30% were men aged between 19 and 34 years old (average age = 27 years) (Figure 2A, B). The functional cookies were rated good for all the sensory attributes. The CS-extract-enriched cookies scored high in terms of overall acceptability (7.60), as well as having the highest ratings in terms of taste (8.00), appearance (7.95), flavor (7.95), sweetness (7.75), crunchiness (7.70), color (7.60), and texture (7.40), placing the cookies between “like moderately” and “like very much” regarding the panelists’ evaluations. Otherwise, a score decay was noticed in terms of hardness (6.25), identifying the cookies between “like slightly” and “like moderately” in terms of hardness. Even though the hardness had the lowest score in terms of the sensory attributes, the score given remained at a fairly good level (score > 5). In general, the acceptance of the functional cookies was 84%, compared to the value of 89% disclosed for the cookies without extract, with all the attributes scored above 5. When compared with the control cookies, the functional cookies disclosed similar or slightly lower scores. All the panelists expressed a desire to acquire the cookies by answering “YES” to the question about purchase intention. Furthermore, the overall acceptability score highlighted the acceptable sensory attributes for the cookies, outlining the positive impact of the CS extract on the sensory properties of the fortified cookies.

All the participants were particularly delighted with the appearance, regarding shape and size, as well as the flavor and taste. Most of the panelists expressed a sensation of a balanced proportion of astringency and bitterness, describing a slightly pleasant bitter and roasting flavor, which was neither too sweet nor too soft. These sensory characteristics were attributed to the phenolic compounds, including the ones identified in the CS extract used to formulate the cookies, such as phenolic acids, flavonoids, and tannins [35,36,37]. For instance, Pedan et al. [37] established a predictive model to investigate the impact of phenolic compounds on the sensory properties of olive oils. Phenolic acids (caffeic, coumaric, ferulic, *p*-hydroxybenzoic, protocatechuic, and vanillic acids), lignans (pinoresinol), and flavones (apigenin and luteolin derivatives) were responsible for the bitterness, pungency, and fruitiness of the olive oils. Recently, Pittari et al. [38] proved that ellagitannins and proanthocyanidins play a pivotal role in the flavor of wine by providing fruitiness, preserving the aroma, and preventing the increase in maderised notes. In this sense, the phenolic acids, flavonoids, hydrolysable tannins, and caffeine identified in the CS-extract-enriched cookies seemed to be responsible for the mild bitterness and astringency described by the panelists. Additionally, only a few evaluators pointed out the dark color of the cookies, which was probably due to the addition of cacao and the CS extract, which had a brownish color. Even though most of the participants appreciated the texture, four panelists indicated the presence of granules and suggested an improvement in the softness to enhance the mouthfeel of the cookies. In addition, the texture score of the cookies may be explained by their high fiber content, as previously reported by Abu-Salem et al. [39] in cookies supplemented with groundnut flour. Three evaluators expressed the high sweetness, which affected their ratings, while the majority found the cookies enjoyable in terms of their sweetness. Concerning crunchiness, only one participant described the cookies as too crunchy. The least-appreciated attribute was hardness when attempting to fracture the cookies, with seven participants giving scores ≤ 5. The present results were in close agreement with the findings of Argyri et al. [27], who attested a good acceptability of cookies enriched with olive pomace (6.86), olive pomace plus garlic, thyme, and oregano (7.55), and olive pomace plus vegetables (7.20) using a nine-point scale. In addition, cookies prepared with chestnut flours from different Italian fruit varieties revealed lower scores (4.38–7.09) in their overall acceptance.

Overall, the evident dark-brownish color of the CS-extract-enriched cookies described by the panelists, along with their mild roasting flavor and slightly bitter aftertaste, did not seem to negatively impact the panel’s sensory perceptions, these even being stated as pleasant characteristics of the product that may positively influence its acceptance by consumers. Considering the contribution of the CS extract to the antioxidant properties of the fortified cookies along with the nutritional value and the acceptable sensory attributes, the promising outcomes sustained the hypothesis that the functional cookies might not only deliver bioactive compounds with pro-healthy effects but also possess extended shelf-life and preservative action owing to the protection against fat oxidation and the antimicrobial properties provided by antioxidants. Hence, CS extract seems to be an innovative and appealing ingredient for a novel formulation of food products, such as cookies.

### 3.6. Correlation Analyses

#### 3.6.1. Phenolic Compounds and Antioxidant/Antiradical Activities

**Figure 3:**
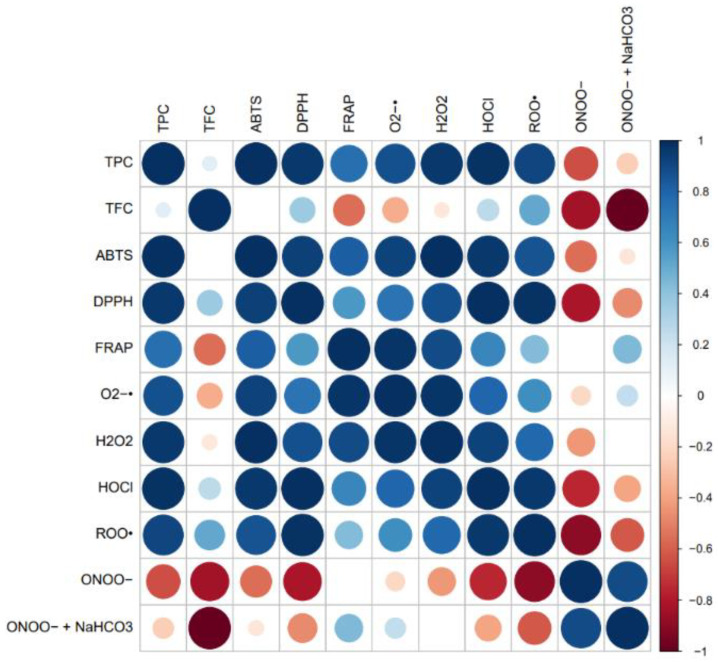
Correlation heatmap between TPC, TFC, antioxidant, and antiradical activity results for functional cookies enriched with chestnut shells extract evaluated by a color diagram.

The Pearson correlations were established to comprehend the impact of the phenolic compounds and flavonoids on the antioxidant and antiradical effects of the cookies enriched with CS extract. In this scenario, a positive correlation would be the desirable outcome. Figure 3 depicts the Pearson correlations between the results of TPC, TFC, antioxidant, and antiradical activities evaluated by a correlation heatmap.

As shown in Figure 3, the TPC analysis achieved very strong positive correlations with ABTS (*r*^2^ = 0.9934), DPPH (*r*^2^ = 0.9690), O_2_^●−^ (*r*^2^ = 0.8794), H_2_O_2_ (*r*^2^ = 0.9686), HOCl (*r*^2^ = 0.9886), and ROO^●^ (*r*^2^ = 0.9156), while a positive correlation was attained between the TPC and FRAP assay (*r*^2^ = 0.7598). These results suggested that an increase in TPC led to enhanced antioxidant responses, proposing the polyphenols from the CS extract as the major bioactive compounds contributing to the antioxidant and antiradical effects of the functional cookies. Oppositely, a moderate negative correlation (*r*^2^ = −0.6474) was reached for the regression analysis between TPC and ONOO^−^ in the absence of NaHCO_3_, indicating that a higher scavenging activity against this reactive species (associated with a lower IC_50_ value) was accomplished by phenolics-rich foods, such as the cookies fortified with the CS extract. Furthermore, the TFC had no correlation or a weak correlation (*r*^2^ ≤ 0.5) with almost all the parameters evaluated, except with ONOO^−^ in the absence and presence of NaHCO_3_, which displayed strong negative correlations (*r*^2^ = −0.8352 and −0.9917, respectively), denoting that a rise in the TFC induced a higher ONOO^−^-quenching power.

The antioxidant activity examined through ABTS radical scavenging attained strong positive correlations with TPC (*r*^2^ = 0.9934), FRAP (*r*^2^ = 0.8295), and DPPH (*r*^2^ = 0.9341), as well as with the scavenging potential against O_2_^●−^ (*r*^2^ = 0.9283), H_2_O_2_ (*r*^2^ = 0.9908), HOCl (*r*^2^ = 0.9647), and ROO^●^ (*r*^2^ = 0.8633). Likewise, the DPPH results disclosed very strong positive correlations with H_2_O_2_ (*r*^2^ = 0.8773), HOCl (*r*^2^ = 0.9951), and ROO^●^ (*r*^2^ = 0.9866), while a strong correlation with O_2_^●−^ (*r*^2^ = 0.7345) and a moderate correlation with FRAP (*r*^2^ = 0.5756) were observed. Conversely, a very strong negative correlation (*r*^2^ = −0.8156) was obtained between the DPPH- and ONOO^−^-quenching activity in the absence of NaHCO_3_. Additionally, very strong positive correlations with FRAP were revealed with O_2_^●−^ (*r*^2^ = 0.9777) and H_2_O_2_ (*r*^2^ = 0.8975), while a moderate correlation was accomplished with HOCl (*r*^2^ = 0.6533). Regarding the biological reactive species, O_2_^●−^ and H_2_O_2_ positively correlated with TPC and almost all the antioxidant/antiradical assays, apart from ONOO^−^ in the absence and presence of NaHCO_3_ (*r*^2^ ≤ 0.5), through very strong (*r*^2^ ≥ 0.9), strong (*r*^2^ ≥ 0.8), and moderate (*r*^2^ ≥ 0.6) effects. HOCl was also positively correlated with almost all the parameters evaluated, including the TPC and in vitro antioxidant activity and radical scavenging assays, except for ONOO^−^ in the absence of NaHCO_3_, which exhibited a strong negative correlation (*r*^2^ = −0.7547). ROO^●^ only disclosed a negative correlation with ONOO^−^ in the absence and presence of NaHCO_3_ (*r*^2^ = −0.8992 and −0.6165, respectively) and no correlation with TFC and FRAP (*r*^2^ ≤ 0.5). Moreover, ONOO^−^ was negatively correlated with almost all the variables, while only a positive correlation was achieved between ONOO^−^ in the absence and presence of NaHCO_3_ (*r*^2^ = 0.8989).

#### 3.6.2. Nutritional Composition, Phenolic Content, and Sensory Properties

**Figure 4:**
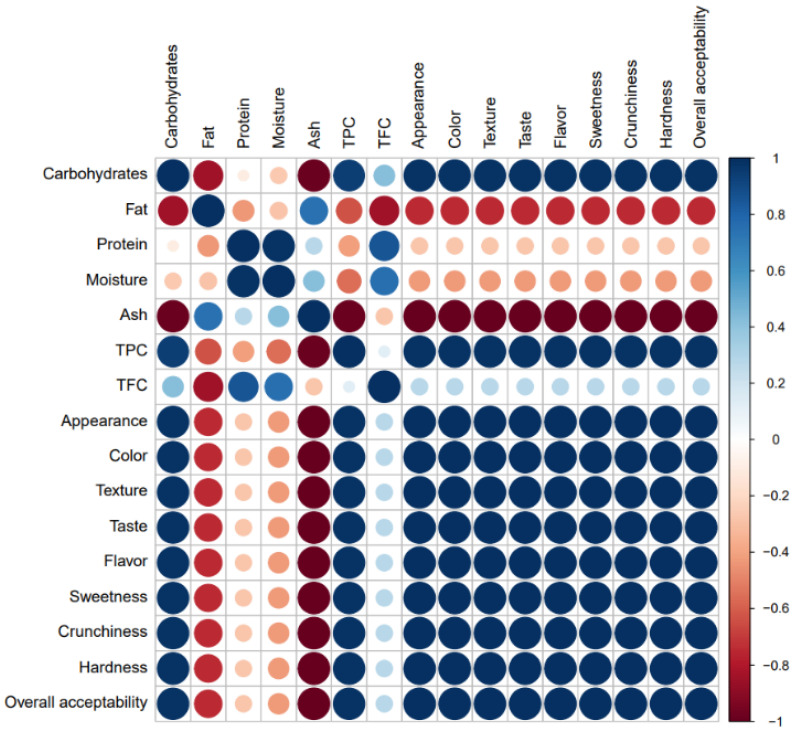
Correlation heatmap between macronutrients, TPC, TFC, and sensory attributes of functional cookies enriched with chestnut shells extract evaluated by a color diagram.

The relationship between the nutritional composition, phenolic content, and sensory properties were assessed through Pearson’s correlations, aiming to explore the contribution of macro and micronutrients to the sensory attributes of the CS-extract-enriched cookies. Figure 4 presents the correlation heatmap using a color diagram.

As depicted in Figure 4, very strong positive correlations were highlighted between carbohydrates and TPC (*r*^2^ = 0.9496), carbohydrates and all sensory attributes (*r*^2^ = 0.9866), protein and TFC (*r*^2^ = 0.8522), and TPC and all sensory attributes (*r*^2^ = 0.9881), suggesting that an increment in one variable led to an increase in the correlated variable. Moreover, fat and ash (*r*^2^ = 0.7488) and moisture and TFC (*r*^2^ = 0.7582) also exhibited strong positive correlations. These results underlined an excellent correlation level, emphasizing an outstanding contribution of carbohydrates and phenolic compounds to the sensory properties of the cookies fortified with CS extract.

Otherwise, negative correlations were found between ash and all the sensory properties (*r*^2^ = −1.0000), carbohydrates and ash (*r*^2^ = −0.9866), ash and TPC (*r*^2^ = −0.9881), carbohydrates and fat (*r*^2^ = −0.8470), fat and TFC (*r*^2^ = −0.8420), fat and all the sensory properties (*r*^2^ = −0.7488), and fat and TPC (*r*^2^ = −0.6377), indicating that an increase in one variable led to a decrease in the respective correlated variable. It is worth noting that a remarkable inverse relation was denoted between ash and fat regarding the sensory attributes, outlining the fact that more appreciated sensory properties were described for the cookies containing low levels of fat and minerals.

Altogether, these correlations seemed to be effective indicators in predicting that high levels of carbohydrates and phenolic compounds, as well as low contents of fat and minerals, were the optimal macro and micronutrient proportions to formulate CS-extract-enriched cookies with the best sensory properties, motivating a better acceptance by consumers.

## 4. Conclusions

This study exploited the use of a nutraceutical extract from CSs, obtained by an eco-friendly technology, as an active ingredient for functional cookies, attempting to pursue a novel approach to valorize this agro-industrial waste. Regardless of the lack of European legislation applied to nutraceuticals, previous studies have validated a new nutraceutical ingredient extracted from CSs by in vitro and in vivo assays following the European Directive 2010/63/EU for animal studies. Nevertheless, this was the first study that attempted to develop functional cookies enriched with a nutraceutical extract from CSs and to appraise their bioactivity, nutritional value, and sensory perception by consumers. The cookies formulated were mainly composed of carbohydrates, fiber, and fat, representing more than 90% dw. Among the micronutrients, the cookies were rich in minerals and phenolic compounds. The antioxidant and antiradical properties were also noteworthy, evidencing the scavenging potential against ROS and RNS, mainly ascribed by gallic acid, ellagic acid, catechin, and caffeoylquinic acids derivatives. Through a correlation analysis, it was possible to demonstrate that the phenolic compounds were the principal compounds responsible for the cookies’ bioactivity. The sensory evaluation accomplished satisfactory results, with the panelists endorsing the acceptability of the product and expressing their desire as potential consumers. Carbohydrates and phenolic compounds were predicted as the main macro and micronutrients, corroborating the most-appreciated sensory properties of the cookies, as outlined by the heatmap correlation. Hence, the current study attested to the promissory health-promoting effects of the functional cookies enriched with a nutraceutical phenolics-rich extract of CSs. Further research should appraise the influence of the in vitro gastrointestinal digestion on the bioaccessibility of the phenolic compounds and estimate their intestinal permeability.

## Figures and Tables

**Table 1:** Formulation of functional cookies enriched with chestnut shells extract prepared by SWE and control cookies (without extract).

Ingredients	Amount (%)	
	Functional Cookies Enriched with CS Extract	Control Cookies (Without Extract)
Sugar	32.20	32.20
White flour	29.40	29.40
Butter	20.70	20.70
Eggs (approx. 2)	7.80	7.80
Cocoa powder	7.08	7.08
Lyophilized chestnut shells extract	1.77	−
Distilled water	−	1.77
Sodium bicarbonate (NaHCO_3_)	0.71	0.71
Sodium chloride (NaCl)	0.34	0.34

**Table 2:** Approximate composition (% dw) of functional cookies enriched with chestnut shells extract and control cookies (without extract).

Approximate Composition	Content (% dw)	
	Functional Cookies Enriched with CS Extract	Control Cookies (Without Extract)
Carbohydrates	53.92 ± 2.67 ^a^	59.73 ± 0.34 ^a^
Fat	32.62 ± 2.83 ^a^	25.04 ± 0.21 ^b^
Protein	6.17 ± 0.57 ^a^	7.59 ± 0.54 ^a^
Fiber	5.15 ± 0.05 ^a^	4.96 ± 0.04 ^a^
Moisture	4.77 ± 0.96 ^a^	3.95 ± 0.08 ^a^
Ash	2.14 ± 0.04 ^b^	2.93 ± 0.13 ^a^
Energy in kJ/100 g (kcal/100 g)	2242.48 (533.92) ^a^	2189.19 (521.24) ^a^

Means with different letters indicate significant differences (*p* < 0.05).

**Table 3:** Total phenolic and flavonoid contents and antioxidant/antiradical activity of functional cookies enriched with chestnut shells extract and control cookies (without extract).

	TPC (mg GAE/100 g Cookies)	TFC (mg CE/100 g Cookies)	ABTS (mg AAE/100 g Cookies)	DPPH (mg TE/100 g Cookies)	FRAP (mg FSE/100 g Cookies)
CS-extract-enriched cookies	163.53 ± 11.00 ^a^	51.49 ± 4.16 ^a^	146.59 ± 8.10 ^a^	67.24 ± 7.04 ^a^	730.16 ± 65.10 ^a^
Control cookies (without extract)	55.89 ± 1.89 ^b^	16.31 ± 1.00 ^b^	58.64 ± 2.79 ^b^	30.56 ± 2.33 ^b^	262.69 ± 3.31 ^b^

AAE, ascorbic acid equivalents. CE, catechin equivalents. CS, chestnut shells. FSE, ferrous sulfate equivalents. FRAP, ferric reducing antioxidant power. GAE, gallic acid equivalents. TE, Trolox equivalents. TFC, total flavonoid content. TPC, total phenolic content. Different letters (a, b, c) denote significant differences between CS-extract-enriched cookies and control cookies (without extract) (*p* < 0.05).

**Table 4:** Counteracting capacity of functional cookies enriched with chestnut shells extract and control cookies (without extract) against reactive oxygen and nitrogen species (*n* = 3).

	Reactive Oxygen Species				Reactive Nitrogen Species	
	O_2_^●−^	H_2_O_2_	HOCl	ROO^●^	ONOO^−^	
	IC_50_ (µg/mL)			Trolox Equivalents (µg TE/mg dw)	In Presence of NaHCO_3_ IC_50_ (µg/mL)	In Absence of NaHCO_3_ IC_50_ (µg/mL)
CS-extract-enriched cookies	35.85 ± 1.04 *^,b^	28.23 ± 0.56 *^,b^	81.81 ± 5.01 ^a^	5.81 ± 0.59 ^c^	108.15 ± 8.45 ^a,1^	115.00 ± 3.59 ^a,1^
Control cookies (without extract)	33.41 ± 1.49 *^,b^	13.39± 1.03 *^,d^	47.00 ± 3.34 *^,b^	0.76 ± 0.05 ^d^	45.56 ± 2.66^*,b,1^	38.48 ± 2.35^*,b,2^
***Positive controls***						
Catechin	48.21 ± 4.79 ^a^	20.78 ± 0.91 ^c^	0.37 ± 0.02 ^d^	368.95 ± 26.39 ^a^	0.23 ± 0.01 ^c,1^	0.16 ± 0.02 ^b,2^
Gallic acid	10.95 ± 1.40 ^c^	106.03 ± 1.14 ^a^	1.81 ± 0.02 ^c^	247.09 ± 51.44 ^b^	0.27 ± 0.04 ^c,1^	0.15 ± 0.02 ^b,2^

IC_50_ corresponds to the in vitro concentration needed to counteract 50% of the reactive species formed in the tested media (mean ± standard error of the mean). Different letters (a, b, c, and d) denote significant differences between extract and positive controls (*p* < 0.05), while different numbers (1 and 2) denote significant differences between the presence and absence of NaHCO_3_ (*p* < 0.05). * Inhibition percentage tested directly in the extract.

**Table 5:** Identification and quantification of phenolic compounds in functional cookies enriched with chestnut shells extract and control cookies (without extract).

Phenolic Compounds	Peak Number	Amount (mg/100 g Cookies)	
		Functional Cookies Enriched with CS Extract	Control Cookies (without Extract)
*Phenolic acids–Hydroxybenzoic acids*			
Gallic acid	1	89.4 ± 4.47 ^a^	36.5 ± 1.83 ^b^
Protocatechuic acid	2	2.02 ± 0.10 ^a^	2.02 ± 0.10 ^a^
Syringic acid	11	0.48 ± 0.02 ^a^	0.13 ± 0.01 ^b^
Vanillic acid	9	0.16 ± 0.01	n.d.
**∑ Hydroxybenzoic acids**		92.06 ^a^	38.65 ^b^
*Phenolic acids–Hydroxycinnamic acids*			
4-*O*-caffeyolquinic acid	8	3.63 ± 0.18 ^a^	3.25 ± 0.16 ^b^
3,4-di-*O*-caffeoylquinic acid	25	1.35 ± 0.07	n.d.
3,5-di-*O*-caffeoylquinic acid	18	0.41 ± 0.02	n.d.
Caffeic acid	10	0.79 ± 0.04	n.d.
Caftaric acid	5	1.28 ± 0.06 ^a^	0.47 ± 0.02 ^b^
Chlorogenic acid	7	2.68 ± 0.13 ^a^	2.22 ± 0.11 ^b^
Neochlorogenic acid	3	1.02 ± 0.05	<LOD
*p*-Coumaric acid	13	0.19 ± 0.01	n.d.
**∑ Hydroxycinnamic acids**		11.35 ^a^	5.94 ^b^
**∑ Phenolic acids**		103.41 ^a^	44.59 ^b^
*Flavanols*			
(+)-Catechin	4	5.17 ± 0.26 ^a^	4.64 ± 0.23 ^b^
(−)-Epicatechin	12	n.d.	3.43 ± 0.17
**∑ Flavanols**		5.17 ^b^	8.07 ^a^
*Flavonols*			
Kaempferol	38	0.068 ± 0.003	n.d.
Kaempferol-3-*O*-glucoside	29	0.81 ± 0.04	n.d.
Kaempferol-3-*O*-rutinoside	31	0.031 ± 0.002	n.d.
Myricetin	26	0.36 ± 0.02	n.d.
Quercetin	35	0.19 ± 0.01	n.d.
Quercetin-3-*O*-galactoside	19	0.33 ± 0.02	n.d.
Quercetin-3-*O*-glucopyranoside	21	0.97 ± 0.05	n.d.
Tiliroside	37	0.018 ± 0.001	n.d.
**∑ Flavonols**		2.78	−
*Flavones*			
Apigenin	39	0.057 ± 0.003	n.d.
Chrysin	40	0.028 ± 0.001	n.d.
**∑ Flavones**		0.085	−
**∑ Flavonoids**		8.04 ^a^	8.07 ^a^
*Hydrolyzable tannins*			
Ellagic acid	24	40.0 ± 2.00	n.d.
**∑ Hydrolysable tannins**		40.0	−
*Others–Alkaloids*			
Caffeine	6	2.71 ± 0.14	n.d.
**∑ Phenolic compounds**		154.09 ^a^	52.66 ^b^

LOD, limit of detection. n.d., not determined. Different letters indicate significant differences (*p* < 0.05) between samples.

## Data Availability

The data used to support the findings of this study can be made available by the corresponding author upon request.
